# The Foot

**Published:** 1916-12

**Authors:** Theodore Toepel

**Affiliations:** Candler Building, Atlanta, Ga.


### Journal-Record of Medicine

Successor to Atlanta Medical and Surgical Journal, Established 1855
and Southern Medical Record, Established 1870

**OWNED BY THE ATLANTA MEDICAL JOURNAL COMPANY**

PUBLISHED MONTHLY

*Official Organ Fulton County Medical Society, State Examing Board, Atlanta, Birmingham and Atlantic Railroad Surgeon's Association, Chattahoochee Valley Medical and Surgical Association, Etc.*

A. W. STIRLING, M. D., C. M., D. P. H.; J. S. HURT, B. Ph., M.D.
GEO. M. NILES, M. D., W. J. LOVE, M. D., (Ala.); Associate Editors

E. W. ALLEN, Business Manager

**COLLABORATORS**

W. F. WESTMORELAND, M. D., General Surgery
F. W. McRAE, M. D., Abdominal Surgery
ALLEN H. BUNCE, Medical Societies
E. B. BLOCK, M. D., Diseases of the Nervous System
MICHAEL HOKE, M. D., Orthopedic Surgery
CYRUS W. STRICKLER, M. D., Legal Medicine and Medical Legislation
E. C. DAVIS, A. B., M. D., Obstetrics
E. G. JONES, A. B., M. D., Gynecology
ALLEN H. BUNCE, M. D., Pathology and Bacteriology
R. T. DORSEY, JR., B. S., M. D., Medicine
L. M. GAINES, A. B., M. D., Internal Medicine
GEO. C. MIZELL, M. D., Diseases of the Stomach and Intestines
L. B. CLARKE, M. D., Pediatrics
EDGAR PAULLIN, M. D., Opsonic Medicine
THEODORE TOEPEL, M. D., Mechano Therapy
E. G. BALLENGER, M. D., Diseases of the Genito-Urinary Organs

VOL. LXIII. ATLANTA, GA., DECEMBER, 1916 No. 9

### THE FOOT.

By Theodore Toepel, M. D., Candler Building, Atlanta, Ga.

In presenting a paper to a body of learned people as are assembled here on a subject which is ordinarily not much discussed by the practicing physician, but which at present gives opportunity for much discussion, I wish to call your attention to some facts that you may have previously known and now forgotten concerning a neglected member of your anatomy, the foot.

I will not describe to you some brilliant operation with the usual success. but I will confine myself to those features in which we are all interested, in the gross anatomy of the normal foot, and some physiology, preventive measures and treatment of easily corrected abnormal conditions.

The bones entering into the formation of the foot are: the astragalus, the os calcis, the scaphoid, the cuboid, the internal, middle and external cuneiforms, the five metatarsals, fourteen phalanges and two sesamoid bones under the head of the first metatarsal.

The ligaments are very numerous between the bones of the foot. I mention the plantar fascia, plantar ligaments, external lateral ligaments; internal lateral ligament, calcaneo-scaphoid ligament and the posterior ligament.

It is well to remember in studying muscles, especially those of the foot, that though the name of the muscle will often indicate one action of which it is capable, it is by no means its only action. Most of the muscles of the foot cross several joints and each of these joints is affectetd by contractions of the muscles crossing it.

In mentioning the muscles contributing to the action of the foot you will readily recall the origin and insertion and necessarily the course of each one of them, the three calf muscles, gastrocnemius, soleus and plantarus, tendo achillis, two peronci, flexor longus pollicis, flexor longus digitorium, accessorius flexor brevis digitorium, Lumbricales, tibialis, posticus, tibialis anticus, extensor proprius pollicio, extensor longus digitorium, pereoneus tertius, abductor pollicis, transversus pedis, the seven interossei and the extensor brevis digitorium.

The nerve supply of all the muscles of the foot comes from two main trunks: the internal and external popliteals.

The physiology of the foot is complex and I will not take up your time in going into the detail description of the various possible movements of the joints that are in the foot but will name only a few of the most important joints,—ankle, subastragaloid, medio tarsal and great toe joint, and be more explicit in describing the physiology of standing and walking.

Were the foot jointless except for the tibio-astragaloid, its physiology would be much simpler than it is with no less than thirty-eight articulations. Many indeed use the foot as though it were one solid mass and more than one surgeon has described it as though its normal movements were limited to flexion and extension at the ankle joint. Were this the case the question of the best standing position would be limited to the best geometrical figure to be formed by the two feet, their distance apart, their relation to each other, and the angle the foot should form with the leg. It is proven beyond doubt that the quadrilateral rather than the trapezoid figure is the best for the feet. That is, we should have the centers of the balls of the feet and the centers of the heels form the corners of a square. If the heels are placed nearly together and the toes turned outward a trapezoid figure is formed, which is decidedly weaker base for support than the square. To bring the transverse plane containing the center of gravity of the body over the strongest part of the bony structures and to keep it there with the least muscular exertion, we should place the foot at about a right angle with the leg. Thus the body of the astragalus would rest mostly on the os calcis. Even with the most careful balancing, however, a condition of absolute equilibrium could be maintained only by having the flexors and extensors in the ankle in almost constant action, correcting the position of the tibia as the gravity-plane was moved backward or forward. This change of weight bearing is easily demostrated by scales: if a stand is erected so as to be contingent to and level with the scale-platform, it will be readily seen that moving the transverse plane ever so slightly changes the proportion of the weight passing through the forefoot and heel. With the subject so placed that the heels are on the stand and the forefoot rests on the scale platform, advancing an arm, nodding the head, contracting the abdominal muscles, raising the chest, will cause an alteration in the reading of the dial. Though a man weighing 150 pounds and standing in low heeled shoes may be said to transmit 100 pounds through the heels and 50 pounds through the forefoot; it must not be supposed that even while one is standing the relation of these two weights is constant. No ligament, muscle or bone is under a constant amount of strain while the body is in the upright position. The sense of balance will cause the plane of the center of gravity to be quickly returned when removed from its normal position; but changes in the amount of strain borne by individual bones, ligaments, and muscles will have occurred, even if momentary, and that is of the greatest importance.

In walking over a moist sandy surface or with wet feet over a dry surface the foot prints remaining upon it will with few exceptions be found turning slightly outward. In the forward motion of the leg the foot is involuntarily turned outward, due to the outward rotation of the hip and knee joints which is caused by the more numerous and stronger abductor muscles against the fewer and weaker abductors. Furthermore upon close observation it will be noticed that during this passive state the outer border of the foot is lower than the inner border. Therefore the progression of the step upon the ground is developed from the heel to the sole in such a manner that the heel touches the ground first whereby in most cases the sole forms an angle of 35o to 45o to the ground. It will be found that the outer border of the heel touches first. Now the outer border of the sole follows the step being further developed toward the inner surface through adjustment of the fanlike spreading of the metatarsal bones toward the second joint of the great toe, from here on to the end of the great toe where the impression is completed and the foot again begins its forward pendulum movement. This development of the impression of the foot explains the wearing of the outer edge of the heel of the shoe and the wearing of the inner edge of the sole, the worn part corresponding to the position of the great toe.

No other member of our body is subjected to distortion and deformity through unsuitable coverings as is the foot and yet it is the foot upon which the greatest demands are placed in supporting and propelling the entire weight of the body. Unfortunately the average customary footwear is impractical. Lectures are given as to proper shoeing of horses in agricultural and veterinary schools but the covering for the human foot is left to the whims of the commercial dealer and to the constant change of styles produced by the designer of fashion. Noting briefly the important features of a comfortable and well fitting shoe which at the same time conforms to the natural shape of the foot, we find that the sole of the shoe must be so shaped that the great toe can rest upon it in its natural relation to the foot. Where this is disregarded ingrown toe-nails and painful bunions result. The uppers must be so shaped at the toes that the free motion of the toes is not interferred with. This is especially necessary over the great toe as we have seen that this member completes the progression in walking. In fitting a shoe it is important to consider comfort in walking and to have the shank fit snugly, thus giving the instep proper support and preventing the foot from slipping forward. The heel must be low and broad. High heels force the foot to an unnatural constant extension and prevent the proper development of the walk from the outer edge of the heel to the great toe, because high heels necessitate walking on the toes alone. This accounts for the frequent turning over of the foot with subsequent sprains.

One of the most common abnormal conditions of which the general practitioner meets is painful feet. As, in the majority of joint conditions, the advice of the orthopedist is sought after the condition becomes chronic, so, too, in painful conditions of the feet, we are not applied to for advice until the condition has gone on for some time and becomes so painful that locomotion is too difficult to be tolerated.

I wish to call attention to the great frequency of foot infections secondary to focal infections in other parts of the body, as in the teeth, gums mouth, throat and nose; also after grip, pneumonia, typhoid fever, etc., these are the cause of the largest percentage of painful feet in adults and children. All physicians will recall such cases where the patients were nurses, policemen, clerks working behind counters and other cases where occupation is one in which constant standing is required,involving all the soft tissues, muscles, tendons, bursae, ligaments, etc. Constant standing with the focal infection causes stretching of the ligaments and breaking down of the arch of the foot which may or may not continue to be painful. Similar conditions develop and are overlooked in children after tonsilitis, measles, scarlet fever, etc. It is therefore well that physicians, when secondary results are looked for in the heart, will investigate the muscles supporting the foot, and when children complain of later rheumatic pains in the foot and legs, proper preventive measures can be used to avoid later chronic foot trouble.

Considering individual cases the proper measures of treatment are as follows: the fixation of the foot and temporary support of the arch by strapping or steel arch supports or by applying a plaster cast which is the most perfect splint, also proper shoeing, corrective exercises or heating by dry air, etc.

The constant wearing of poorly fitted and unhygienic foot wear as described heretofore produces fallen arches, backaches, headaches, general nervous conditions, lordosis, displacement of the visera and reproductive organs.

In seeking remedies for these ills the conscientious physician instead of prescribing medicines will effect more lasting and beneficial results when he first condemns ill fitting and high heel shoes by his patients and endeavors by education to make popular, especially among his women patients, a sane and hygienic foot wear.

Read before the Chattahoochee Valley Medical Association, Alexander City, Ala.

